# Male partner antenatal clinic attendance is associated with increased uptake of maternal health services and infant BCG immunization: a national survey in Kenya

**DOI:** 10.1186/s12884-019-2438-9

**Published:** 2019-08-08

**Authors:** Beryne Odeny, Christine J. McGrath, Agnes Langat, Jillian Pintye, Benson Singa, John Kinuthia, Abraham Katana, Lucy Ng’ang’a, Grace John-Stewart

**Affiliations:** 1 Department of Global Health, University of Washington, 325 9th Ave #359909, Seattle, WA USA; 2 Department of Nursing, University of Washington, Health Sciences Building, T-301, 1959 NE Pacific St, Seattle, WA USA; 3 Department of Medicine, University of Washington, Health Sciences Building, RR-512, 1959 NE Pacific St, Seattle, WA USA; 4 Department of Epidemiology, University of Washington, Health Sciences Building, F-262, 1959 NE Pacific St, Seattle, WA USA; 5 United States Centers for Disease Control and Prevention (CDC), P.O. Box 606-00621, Village Market, Nairobi, Kenya; 6 Center for Microbiology Research and Center for Clinical Research, Kenya Medical Research Institute, P.O. Box 19464-00202, Nairobi, Kenya; 7 Department of Research & Programs, Kenyatta National Hospital, P.O. Box 20723-00202, Nairobi, Kenya

**Keywords:** Maternal child health, Antenatal care, Male partner, Attendance, Involvement, HIV

## Abstract

**Background:**

Male partner antenatal clinic (ANC) attendance may improve maternal uptake of maternal child health (MCH) services.

**Methods:**

We conducted a cross-sectional survey of mother-infant pairs attending week-6 or month-9 infant immunizations at 120 high-volume MCH clinics throughout Kenya. Clinics were selected using probability proportionate to size sampling. Women were interviewed using structured questionnaires and clinical data was verified using MCH booklets. Among married women, survey-weighted logistic regression models accounting for clinic-level clustering were used to compare outcomes by male ANC attendance and to identify its correlates.

**Results:**

Among 2521 women attending MCH clinics and had information on male partner ANC attendance, 2141 (90%) were married of whom 806 (35%) had male partners that attended ANC. Among married women, male partner ANC attendance was more frequent among women with higher education, women who requested their partners to attend ANC, had male partners with higher education, did not report partner violence, and had disclosed their HIV status (*p* < 0·001 for each). Additionally, male ANC attendance was associated with higher uptake of ANC visits [adjusted Odds Ratio (AOR) = 1·67, 95% confidence interval (CI) 1·36–2·05,], skilled delivery (AOR = 2·00, 95% CI 1·51–2·64), exclusive breastfeeding (AOR = 1·70, 95% CI 1·00–2·91), infant Bacille Calmette Guerin (BCG) immunization (AOR = 3·59, 95% CI 1·00–12·88), and among HIV-infected women, antiretroviral drugs (aOR = 6·16, 95% CI 1·26–30·41).

**Conclusion:**

Involving male partners in MCH activities amplifies benefits of MCH services by engaging partner support for maternal uptake of services.

## Background

Maternal and child health (MCH) services are widely available in sub-Saharan Africa, however, maternal and infant mortality and morbidity remain unacceptably high [1, 2]. Access to MCH services is a first step to receiving antenatal care (ANC), postnatal care (PNC), family planning, infant immunizations, growth monitoring, and prevention of mother-to-child transmission of HIV (PMTCT) interventions and is key to healthy maternal and infant outcomes [3]. World Health Organization (WHO) guidelines recommend that pregnant women attend at least 4 ANC visits as part of focused antenatal care (FANC) [1]. However, uptake of FANC and other MCH services among Kenyan women is low (< 90%) [2–4].

In Kenya, as in other low resource settings, socioeconomic and cultural factors limit women’s autonomy to access maternal and infant health services [2, 5, 6]. Women remain vulnerable to gender inequality norms and economic constraints that increase their dependence on men and undermine their independent access to MCH services [5, 6]. As a result, decision-making that affects women’s and children’s health often rests on male partners [7]. Understanding the role played by men and the need to involve male partners in MCH services has become more widely recognized [7, 8]. Male ANC attendance has been recognized as a strategy for male involvement in MCH [9–11]. Enabling men to visit ANC provides an opportunity to educate men on maternal and child health while fostering their favorable attitudes toward access of services [12, 13]. There is some evidence to suggest that male ANC attendance may enhance access to skilled deliveries, antenatal care, and use of antiretroviral drugs (ARVs) for PMTCT [6, 10, 11, 14–18]. Unfortunately, male partner ANC attendance is often low and efforts to encourage it may be suboptimal [15, 19, 20].

As part of a national survey, we evaluated the prevalence and correlates of male ANC attendance and its association with maternal uptake of antenatal and PMTCT services among married, postpartum women.

## Methods

### Study design and sampling framework

**Fig. 1:**
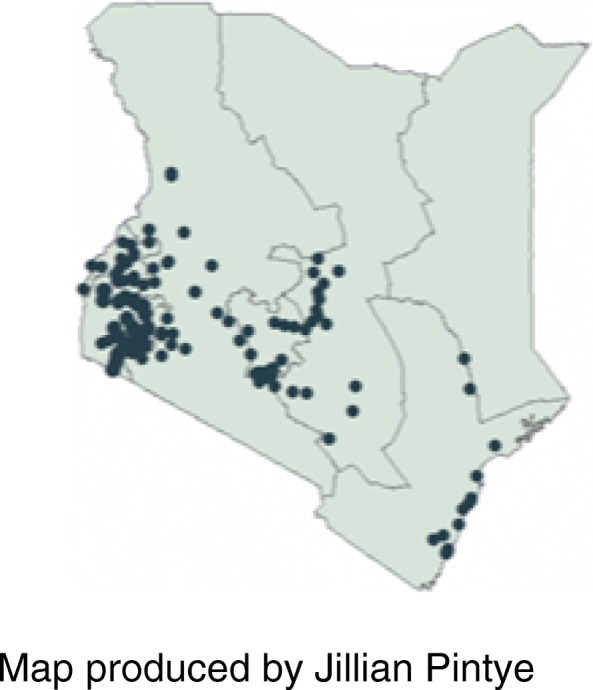
Sites surveyed across Kenya. Map produced by Jillian Pintye

We conducted a cross-sectional facility-based survey in MCH clinics throughout Kenya between June and December 2013 to assess coverage and uptake of antenatal and PMTCT services. MCH clinics that provided routine MCH, including PMTCT services, were included in the sampling frame. Facilities in Wajir, Mandera and Garissa counties and facilities with < 500 ANC visits in 2011, as reported to the National AIDS and STI Control Programme (NASCOP), were excluded from the sampling frame due to logistical constraints. This sub-analysis includes data from the primary PMTCT-MCH survey which used probability proportionate to size sampling to randomly select 120 of 540 facilities with ≥500 ANC visits per year in 44 of 47 counties in Kenya (Fig. 1).

### Participant eligibility and study procedures

Trained study teams, consisting of one nurse and one laboratory technician, collected data from eligible participants during a 5-day window per clinic. Women were eligible if they were the biological mothers bringing their infants for routine week 6 or month 9 infant immunizations and were willing and able to provide written informed consent.

Staff-administered structured questionnaires were used to gather information on male ANC attendance and maternal and partner sociodemographic characteristics as reported by the mother. Partner characteristics included age, level of education, employment status, HIV status, and provision of financial support. Maternal sociodemographic characteristics included age, marital status, relationship duration, education level, employment status, partner support, maternal orphan status, and intimate partner violence (IPV). IPV was assessed using the four-items: Hurt, Insulted, Threatened with Harm, and Screamed (HiTS) Domestic violence screening tool, measuring the frequency a partner hurts, insults, threatens or screams [21]. A ‘yes’ to any of the four items was defined as presence of IPV. Sharing a toilet with another household was used as a proxy measure of socioeconomic status. Clinical information included maternal and infant HIV status, gestational age at first ANC visit, number of ANC visits, and uptake of ARVs for PMTCT. MCH booklets were used to confirm maternal ANC attendance, skilled delivery, ARVs for PMTCT, and infant BCG immunization.

All participants provided written informed consent prior to enrollment in the study. Ethical approvals were obtained from the University of Washington Human Subjects Division, the Kenya Medical Research Institute, and the Associate Director for Science at the U.S. Centers for Disease Control and Prevention (CDC).

### Statistical analysis

Analyses were restricted to married women who had male partner ANC attendance data to control for unmeasured effects of relationship type and stability on male ANC attendance. A woman was considered to be married, if she reported that she was in a monogamous or polygamous marriage or was in a “come-we-stay” relationship with her male partner. Only married women with information on male partner ANC attendance were analyzed. We conducted analyses to estimate prevalence and correlates of male ANC attendance, and determine associations between male ANC attendance and maternal and infant health outcomes among married women. Chi-square tests were used to compare characteristics of the mother and her male partner by male ANC attendance. Logistic regression with odds ratios (OR), adjusted ORs (AOR), and 95% confidence intervals (CI) were used to analyze covariates. Continuous data were analyzed and presented using medians and interquartile ranges. Male ANC attendance was also compared by maternal HIV status. All estimates were weighted to reflect probability for selection of each study clinic and adjusted for clustering at the clinic level. Age and education were modeled as continuous and ordinal variables, respectively. Maternal age and education were included in multivariable logistic regression a priori assumptions based on their known associations with uptake of maternal health services. All analyses were conducted using Stata 13·0 *svy* commands (STATA Corporation, College Station, Texas).

## Results

### Study population

Of 2521 mother-infant pairs enrolled, analyses were done on 2141 women (90%) who were married and had partner attendance data. Their median age was 25 years (Interquartile range (IQR) 22–30 years) and adolescent mothers aged 10–19 years, accounted for 10% (*n* = 173) of married women (Table 1). Forty four percent (*n* = 974) of married women had attained at least secondary level education and 36% (*n* = 808) were employed. Forty-one percent (*n* = 894) of married women experienced some form of IPV. Sixty three percent (*n* = 1346) of married women reported they had at least one living parent and 58% (*n* = 1279) reported sharing a toilet with another household. Among married women, 7% (*n* = 153) were HIV-positive.

**Table 1:** Maternal correlates for male partner ANC attendance among married women^a^

	Unweighted N (Weighted %) or Weighted Median (IQR)						
	Partner attended ANC						
	Total^c^ (*n* = 2141)	No (*n* = 1335)	Yes (*n* = 806)	OR (95% CI)	*P*-value	AOR (95% CI)^b^	*P*-value
HIV positive	158 (7)	106 (7)	52 (7)	1·05 (0·69–1·59)	0·811	1·21 (0·66–2·24)	0·537
Median age, years	25 (22–30)	25 (21–30)	24 (22–29)	0·98 (0·96–0·99)	0·046	0·97 (0·95–0·99)	0·006
Requested partner to attend ANC							
No	774 (41)	711 (58)	63 (9)	Reference		Reference	
Yes	1366 (59)	623 (42)	743 (91)	14·16 (10·09–19·87)	< 0·001	13·31 (9·21–19·21)	< 0·001
Maternal education							
≤ Primary	1167 (56)	830 (67)	337 (45)	Reference		Reference	
Secondary	678 (31)	382 (25)	296 (37)	2·26 (1·77–2·87)	< 0·001	2·26 (1·77–2·88)	< 0·001
> Secondary	296 (13)	123 (8)	173 (18)	3·5 (2·58–4·94)	< 0·001	3·82 (2·75–5·31)	< 0·001
Employed							
No	1311 (64)	849 (66)	462 (60)	Reference		Reference	
Yes	808 (36)	468 (34)	340 (40)	1·33 (1·07–1·66)	0·012	1·21 (0·94–1·56)	0·152
Disclosed HIV status to partner	2036 (96)	1245 (93)	791 (98)	3·28 (1·60–6·75)	0·001	3·07 (1·41–6·67)	0·005
Among HIV positive	135 (86)	88 (81)	47 (90)	1·82 (0·53–6·27)	0·342	1·84 (0·66–5·08)	0·241
Among HIV negative	1900 (97)	1156 (94)	744 (99)	4·07 (1·61–10·33)	0·003	3·64 (1·46–9·04)	0·006
Has other male partners	78 (3)	49 (3)	29 (3)	1·08 (0·63–1·84)	0·776	1·03 (0·57–1·88)	0·916
Intimate partner violence							
No	1247 (59)	717 (52)	530 (67)	Reference		Reference	
Yes	894 (41)	618 (48)	276 (33)	0·55 (0·44–0·68)	< 0·001	0·61 (0·46–0·79)	< 0·001
Not orphaned	1346 (63)	831 (63)	515 (63)	1·02 (0·82–1·27)	0·871	0·87 (0·69–1·10)	0·140
Shared toilet							
No	862 (42)	507 (40)	355 (44)	Reference		Reference	
Yes	1279 (58)	828 (60)	451 (56)	0·72 (0·58–0·89)	0·003	0·73 (0·57–0·95)	0·017

^a^All estimates account for sampling design and clinic-level clustering

^b^Multivariable model adjusting for maternal age and maternal education

^c^Married women without partner attendance data were excluded from the analysis

Median age of married male partners was 30 years (IQR 26–35 years) (Table 2). Sixty percent of men had attained at least secondary school education (*n* = 1257), 87% had some form of employment (*n* = 2142), and 98% (*n* = 2165) provided financial support to their spouses. Among 77% (*n* = 1509) of women who reported knowing their spouses’ HIV status, 4% (*n* = 76) of male partners were HIV-positive.

**Table 2:** Paternal Correlates of Male ANC attendance^a^

	Unweighted N (Weighted %) or Weighted Median (IQR)						
	Partner attended ANC (35%)						
	Total^c^ (n = 2141)	No (n = 1335)	Yes (n = 806)	OR (95% CI)	*p*-value	AOR (95% CI)^b^	*p*-value
Median age, years	30 (26–35)	30 (26–37)	30 (27–35)	0·97 (0·96–0·99)	< 0·001	0·97 (0·96–0·99)	0·002
Age, categorized							
18–25 years	209 (11)	135 (12)	74 (10)	Reference		Reference	
25–35 years	1090 (54)	607 (45)	483 (62)	1·57 (1·08–2·30)	0·019	1·40 (0·96–2·06)	0·082
> 35 years	696 (35)	501 (43)	195 (28)	0·76 (0·51–1·14)	0·186	0·84 (0·53–1·34)	0·466
Education							
≤ Primary	786 (40)	564 (49)	222 (31)	Reference		Reference	
Secondary	873 (43)	535 (40)	338 (45)	1·78 (1·38–2·29)	< 0·001	1·42 (1·06–1·90)	0·018
> Secondary	384 (17)	164 (11)	220 (24)	3·24 (2·38–4·40)	< 0·001	1·90 (1·24–2·85)	0·003
Employed	2142 (87)	1162 (85)	713 (88)	1·26 (0·89–1·78)	0·193	1·11 (0·79–1·55)	0·558
Provides financial support	2165 (98)	1297 (97)	794 (99)	2·37 (0·77–7·26)	0·132	2·28 (0·64–8·14)	0·206
Partner tested for HIV	1509 (77)	812 (69)	682 (91)	4.32 (3.12–5.99)	< 0.001	3.67 (2.63–5.13)	< 0.001
HIV positive	76 (4)	47 (4)	29 (4)	0·98 (0·57–1·70)	0·948	1·20 (0·70–2·07)	0·514
Median relationship duration, years	4 (2–8)	5 (2–10)	3 (1·5–6)	0·92 (0·90–0·95)	< 0·001	0·92 (0·89–0·95)	< 0·001
Median number of children	3 (2–4)	3 (2–4)	2 (2–3)	0·75 (0·67–0·83)	< 0·001	0·76 (0·66–0·88)	< 0·001

^a^All estimates account for sampling design and clinic-level clustering

^b^Multivariable model adjusting for maternal age and maternal education

^c^Married women without partner attendance data were excluded from the analysis

### Prevalence and correlates of partner attendance

Thirty five percent of male partners attended ANC (Table 2). Older women were less likely to have their male partners attend ANC. The likelihood of male ANC attendance lowered with each increasing year of maternal age [AOR = 0·97 (0·95–0·99), *P* = 0·006]. Male partner ANC attendance was less likely if there was a history of IPV [AOR = 0·61 (0·46–0·79), *p* < 0·001] and if women reported their households shared a toilet [AOR = 0·73 (0·57–0·95), *p* = 0·017] (Table 1). Conversely, male ANC attendance was associated with maternal disclosure of HIV status [AOR = 3·07 (1·41–6·67), p = 0·005]. Women who completed at least secondary school were more than two times as likely to have their male partners attend ANC compared to those with primary or no education [AOR = 2·26 (1·77–2·88), *p* < 0.001]. Male partner attendance was 13 times higher among men asked to attend clinic by their spouses than those not asked not to attend [AOR = 13·31 (9·21–19·21), p < 0·001]. Women with secondary education [AOR = 1.77 (1.42–2.22), *p* < 0.001] and higher than secondary education [AOR = 2.97 (2.14–4.11), *p* < 0.001] were twice as likely to request their partners to come to ANC compared to women with primary or no education (Table 3). Positive maternal HIV status was also associated with a significantly higher likelihood of women requesting their partners to attend ANC [AOR = 1.79 (1.11–2.90), *p* = 0.017] (Table 3).

**Table 3:** Characteristics of married women who requested their partners to attend ANC^a^

	Unweighted N (Weighted %) or Weighted Median (IQR)						
	Requested Partner to attend ANC						
	Total^c^ (n = 2141)	No (n = 1335)	Yes (n = 806)	OR (95% CI)	*P*-value	AOR (95% CI)^b^	*P*-value
HIV positive	148	42	116	1.67 (1.05–2.65)	0.029	1.79 (1.11–2.90)	0.017
Median age, years	25 (22–30)	25 (21–30)	25 (22–29)	0.99 (0.98–1.02)	0.905	0.99 (0.98–101)	0.365
Maternal education							
≤ Primary	1167 (60)	499 (67)	667 (45)	ref		ref	
Secondary	678 (29)	213 (25)	465 (37)	1.77 (1.42–2.22)	< 0.001	1.77 (1.42–2.22)	< 0.001
> Secondary	296 (11)	62 (8)	234 (18)	2.91 (2.10–4.04)	< 0.001	2.97 (2.14–4.11)	< 0.001
Employed							
No	1312 (64)	490 (67)	820 (44)	ref		ref	
Yes	809 (36)	275 (33)	533 56)	1.23 (0.92–1.64)	0.162	1.09 (0.80–1.50)	0.569
Has other male partners							
No	2052 (97)	740 (97)	1312 (97)	ref		ref	
Yes	78 (3)	30 (3)	48 (3)	0.94 (0.55–1.61)	0.816	0.91 (0.53–1.58)	0.750
Intimate partner violence							
No	1247 (57)	450 (56)	797 (59)	ref		ref	
Yes	894 (43)	324 (44)	569 (41)	0.89 (0.70–1.13)	0.327	0.97 (0.77–1.23)	0.830

^a^All estimates account for sampling design and clinic-level clustering

^b^Multivariable model adjusting for maternal age and maternal education

^c^Married women without partner attendance data were excluded from the analysis

Male partners were more likely to attend ANC if they completed secondary school [AOR = 1·42 (1·06–1·90), *p* = 0·018] or college [AOR = 1·90 (1·24–2·85), *p* = 0·003] as compared to men with less education. Older men were less likely to attend ANC than younger men [AOR = 0·97 per increasing year of age (0·96–0·99), *p* = 0·002], and male partners with multiple children were less likely to attend ANC than male partners with fewer children [AOR = 0·76 (0·66–0·88), *p* < 0·001]. Additionally, male partners in longer duration relationships were less likely to accompany their spouses to ANC compared to those in more recent relationships [AOR = 0·92 per increasing year (0·89–0·95), *p* < 0·001] (Table 2). Male partner employment, provision of financial support, and HIV status were not associated with ANC attendance.

### Maternal uptake of MCH and PMTCT services and male ANC attendance

After adjusting for maternal age and education and clustering at the clinic level, women whose male partners attended MCH were more likely to attend ≥4 ANC visits during pregnancy than women with partners that did not attend MCH [AOR = 1·67 (1·36–2·05), *p* < 0·001]. Facility delivery was 2 times higher among women whose partners attended ANC [AOR = 2·00 (1·51–2·64), p < 0·001], and the prevalence of exclusive breastfeeding at 6 weeks postpartum was higher among women whose partners attended ANC [AOR = 1·70 (1·00–2·91), *p* = 0·051]. There was a 3 times higher likelihood of Bacille Calmette Guerin (BCG) immunization among infants whose fathers attended ANC compared to infants whose fathers did not attend ANC [AOR = 3·59 (1·00–12·88), p = 0·050] (Table 4)*.*

**Table 4:** Male ANC attendance and maternal-infant health outcomes among married women^a^

		Unweighted N (Weighted %) or Weighted Median (IQR)					
		Partner attended ANC					
Married women^f^	Total n^f^ (n = 2141)	No (n = 1335)	Yes (n = 806)	OR (95% CI)	*p*-value	AOR (95% CI)^b^	*p*-value
ANC visits	2141						
≥ 4 ANC visits		556 (40)	468 (57)	1·98 (1·59–2·48)	< 0·001	1·67 (1·36–2·05)	< 0·001
< 4 ANC visits		731 (60)	306 (43)				
Not recorded		48	32				
Skilled delivery	2141						
Yes		976 (68)	700 (85)	2·62 (1·97–3·50)	< 0·001	2·00 (1·51–2·64)	< 0·001
No		359 (32)	106 (15)				
Not recorded		0	0				
6-week Exclusive breastfeeding among women attending week 6 visit^c^	1278						
Yes		718 (90)	453 (95)	1·98 (1·07–3·69)	0·031	1·70 (1·00–2·91)	0·051
No		76 (10)	31 (5)				
Not recorded		0	0				
6-month Exclusive breastfeeding among women attending month 9 visit^d^	863						
Yes		390 (70)	242 (74)	1·18 (0·80–1·75)	0·397	1·08 (0·75–1·54)	0·680
No		151 (30)	80 (26)				
Not recorded		0	0				
HIV+ married women	*n* = 158						
ARVs for PMTCT (self-report)	158						
Yes		49 (72)	33 (95)	7·05 (1·51–32·84)	0·013	6·16 (1·26–30·41)	0·026
No		11 (28)	3 (5)				
Not recorded		46	16				
ARVs for PMTCT (verified in MCH book)	158						
Yes		33 (61)	19 (92)	7·02 (1·41–34·89)	0·018	5·50 (0·99–30.40)	0·051
No		11 (39)	3 (8)				
Not recorded		62	30				
All Infants	Total n	n = 1335	n = 806	OR (95% CI)	p-value	AOR (95% CI)^a^	p-value
Infant BCG^e^ immunization	2141						
Yes		1317 (98)	803 (100)	4·45 (1·23–16·09)	0·023	3·59 (1·00–12·88)	0·050
No		18 (2)	3 (0)				
Not recorded		0	0				
HIV-exposed infants	Total n						
Infant received ARVs for PMTCT	158						
Yes		99 (98)	48 (95)	0·34 (0·05–2·33)	0·270	0·30 (0·04–2·18)	0·225
No		4 (2)	3 (5)				
Not recorded		3	1				
Infant had a HIV PCR test	158						
Yes		51 (43)	26 (53)	1·53 (0·69–3·39)	0·292	1·59 (0·73–3·47)	0·241
No		29 (57)	16 (47)				
Not recorded		26	10				

^a^All estimates account for sampling design and clinic-level clustering

^b^Multivariable model adjusting for maternal age and maternal education

^c^Measured among women attending week-6 postpartum visit, and adjusted for maternal HIV status

^d^Measured among women attending month-9 postpartum visit

^e^Bacille Calmette Guerin

^f^Married women without partner attendance data were excluded from the analysis

Among 158 HIV-infected women who had partner ANC attendance data, male ANC attendance was associated with a significantly higher prevalence of ARV use for PMTCT; both self-reported [AOR = 6·16 (1·26–30·41), p = 0·026] and as verified in the MCH booklet [AOR = 5·50 (0·99–30.40), p = 0·051] (Table 4).

## Discussion

In this geographically dispersed facility-based survey in Kenya, we found a relatively high prevalence of partner ANC attendance with more than one-third of male partners attending at least one ANC visit. Male ANC attendance was associated with a higher prevalence of maternal ANC attendance, facility delivery, exclusive breastfeeding, infant BCG immunization, and uptake of ARVs. To our knowledge this is the first study to provide national estimates of male ANC attendance in Africa. The estimated national prevalence of male ANC attendance in this survey was 33% overall and 35% among married women, respectively. In smaller regional studies from Eastern Uganda and Northern Ethiopia, male partner ANC attendance ranged from 5 to 20% [22, 23]. While male ANC attendance in this survey is higher than in previous studies, there remains considerable room for improvement [15, 20, 24].

Men’s attitudes, perceptions, and understanding of MCH services influences their support of maternal access to these services [7]. Thus, male education is a gateway to increasing male support of maternal engagement with MCH services [13, 25–28]. A recent systematic review demonstrated that men’s motivation to ensure a safe delivery is an important outcome of male ANC attendance [29]. Approaches to encourage male ANC attendance include use of participatory exercises with men to discuss a father’s supportive role [30]. A hospital-based study in Malawi reported a 2-year increase in male partner ANC attendance from less than 1% to 10·7% following implementation of peer education, peer-led male-involvement drama, and male-friendly hospital infrastructure [31]. Provision of more convenient clinic attendance options, abbreviated male check-up, calling or visiting men who cannot make it to ANC clinic and HIV testing are opportunities for initiating conversations around maternal health [13–15, 32, 33].

We found that a woman having a male partner who was younger and more educated and having fewer children increased the male partner’s likelihood of attending ANC. Among married women, being younger, higher education, absence of partner violence, and higher socioeconomic status were associated with male ANC attendance. Male partners in longer duration relationships were significantly less likely to attend ANC. Similarly, previous studies have shown that marital status, employment, awareness of ANC benefits, male perceptions, living with female partner, distance from facility, and intimate partner violence significantly affect male ANC attendance [9, 20, 30, 34].

Analysis of self-reported information revealed that mothers’ simply requesting their partners to attend ANC increased their partners’ likelihood of attending ANC 13-fold. Additionally, higher maternal education was associated with higher chances of requesting partners to attend ANC. While male perceptions are important determinants of ANC attendance, women’s perceptions and initiative to request partners to accompany them to ANC are significantly influential. This is a significant finding as MCH programs could increase male partner involvement by equipping women with the skills and tools necessary to invite or request their male partners to attend ANC [35]. Partner invitation to clinic could potentially be important for those men in longer duration relationships who may be less likely to attend clinic. This is consistent with findings from studies in Tanzania, South Africa, and Ethiopia in which invitation of partners catalyzed their ANC attendance [12, 22, 35].

In Kenya, widespread campaigns have been used to encourage male partner engagement in MCH activities [10, 17, 30, 32]. Regional efforts are primarily driven by non-governmental organizations (NGOs) [18, 30, 32, 36]. Population Services International (PSI) conducted campaigns aimed at increasing male involvement in PMTCT and reported an increase in partner support following campaigns [36]. The “Tunza” family health network by PSI promoted male involvement and support for maternal contraception use [36]. ICAP at Columbia University, Kenya’s “Egemesha Wanawake Program” was an intervention targeting male partners’ knowledge and skills to support family access to MCH services [30]. The Family AIDS Care and Education Services (FACES) initiative aimed at increasing male ANC attendance, reported increase from 7·4% to 54·2% male attendance among 7236 women [32]. We posit that the relatively high prevalence of male partner attendance that we observed may reflect these efforts and demonstrates significant progress in Kenya [14, 15]. In Rwanda, men have been encouraged to become involved in pregnancy and PMTCT care following a government initiative [37]. In Kenya, more remains to be done with regard to creating national policies that support partner ANC attendance efforts.

In our study, male ANC attendance was associated with uptake of multiple health services including maternal adherence to 4 or more ANC visits, facility delivery, and infant BCG immunization. Globally, access to facility delivery alone can reduce maternal mortality by 16–33% [38]. We also found that women whose male partners attended clinic and were engaged in their care were more likely to exclusively breastfeed. The health of a child depends on that of the mother, and delivery of effective MCH interventions to women could avert up to 40% of deaths in children under five [39]. HIV-infected women were also more likely to use ARVs for PMTCT if their partners attended ANC. In prior studies from Kenya and South Africa, couple-based PMTCT interventions increased uptake of PMTCT [14, 15, 17, 36, 40, 41]. We did not detect differences in partner attendance between HIV-infected and HIV-uninfected women, suggesting that married HIV-infected women may access partner support as readily as married HIV-uninfected women.

While male ANC attendance appears to confer many benefits, some women cannot access this support in the absence of a stable partnerships or when in abusive relationships [30]. In our survey, 41% of women were in abusive partnerships. These women may need tailored interventions to motivate their uptake of services. In addition, male ANC attendance could sometimes lead to negative outcomes [42]. HIV disclosure to partners may result in post-test domestic violence or abandonment [30, 42]. Due to the cross-sectional nature of our survey, we cannot determine the causal direction between IPV and male attendance. In other studies, IPV has been associated with low uptake of facility delivery and a higher likelihood of morbidity in pregnancy [36, 43, 44]. Our findings suggest that IPV, which is a potential barrier to male support and maternal access to services, should be anticipated and addressed in health programs. Where possible, couples should be linked to domestic violence programs, counselling interventions, and support groups to improve the way couples handle disclosure of important information [30, 42].

A primary strength of our study is the large geographically dispersed sample of MCH clinics throughout Kenya. Multivariate analysis allowed us to adjust for potential confounders such as age and education, though there could be residual confounding. We used in-person interviews and validated self-report with maternal and infant records. Mother-infant pairs were selected based on attendance at week-6 and month-9 infant immunizations, both of which are highly attended. Our study has limitations given that data could have been influenced by social desirability bias as we depended on maternal verbal reports. Another limitation is the selection bias with regards to examining infant BCG immunization in that participants were selected from women bringing their infants for routine week 6 or month 9 infant immunizations. Populations coming for immunization are different from those that don’t. This could potentially reduce the power to detect a difference between women whose partners attended ANC and those whose partners did not. While we found that male partner ANC attendance was associated with increased likelihood of BCG immunization uptake and ARV use for PMTCT, the confidence intervals are wide indicating that estimates should be interpreted with caution. However, the results are consistent with other published literature that report a positive association between male partner involvement and maternal and child health outcomes.

## Conclusion

Our study demonstrates associations between male partner ANC attendance and maternal ANC visits, facility delivery, exclusive breastfeeding, infant BCG immunization, and maternal ARV use. Male partner ANC attendance may be useful to accelerate progress to achieving better maternal and infant health outcomes. It is possible to increase male partner ANC attendance regardless of HIV status. This strategy should to be pursued given the benefits conferred to maternal child health. Concerted programmatic efforts are needed to support mothers in inviting their partners to ANC, with special considerations for women who experience intimate partner violence and those of low socioeconomic status for whom engaging male partners may be especially challenging.

## Data Availability

The datasets used and/or analyzed during the current study are available from the corresponding author on reasonable request.

## Abbreviations

ANC: Antenatal Care
ART: Antiretroviral Therapy
ARVs: Antiretroviral drugs
BCG: Bacille Calmette Guerin
CDC: U.S. Centers for Disease Control and Prevention
CI: Confidence Intervals
FACES: Family AIDS Care and Education Services
FANC: Focused Antenatal Care
HiTS: Hurt, Insulted, Threatened with harm, and Screamed Domestic Violence screening tool
HIV: Human Immunodeficiency Virus
IPV: Intimate Partner Violence
KEMRI: Kenya Medical Research Institute
MCH: Maternal and Child Health
NASCOP: National AIDS and STI Control Program
NGO: Non-Governmental Organization
OR: Odds Ratio
PCR: Polymerase Chain Reaction
PMTCT: Prevention of Mother-to-Child Transmission of HIV
PNC: Postnatal Care
PSI: Population Services International
WHO: World Health Organization
